# Induced abortion: a cross-sectional study on knowledge of and attitudes toward the new abortion law in Maputo and Quelimane cities, Mozambique

**DOI:** 10.1186/s12905-020-00988-6

**Published:** 2020-06-19

**Authors:** Mónica Frederico, Carlos Arnaldo, Peter Decat, Adelino Juga, Elizabeth Kemigisha, Olivier Degomme, Kristien Michielsen

**Affiliations:** 1 Centro de Estudos Africanos, Eduardo Mondlane University, Maputo, Mozambique; 2 International Centre for Reproductive Health (ICRH), Department of Public Health and Primary Care, Faculty of Medicine and Health Sciences, Ghent University, Ghent, Belgium; 3 Department of Family Medicine and Primary Health Care, Ghent University, Ghent, Belgium; 4 Department of Mathematics and Informatics, Faculty of Sciences, Eduardo Mondlane University, Maputo, Mozambique; 5 I-BioStat, Hasselt University, Diepenbeek, Belgium; 6 Faculty of Interdisciplinary Studies, Mbarara University of Science and Technology, Mbarara, 1410 Uganda

**Keywords:** Induced abortion, Abortion legislation, Women, Maputo, Quelimane, Knowledge

## Abstract

**Background:**

Maternal mortality, of which 6.7% is attributable to abortion complications, remains high in Mozambique. The objective of this paper is to assess the level of induced abortion at the community, as well as to assess awareness of and attitudes towards the new abortion law among women of reproductive age in suburban areas of Maputo and Quelimane cities.

**Methods:**

A cross-sectional household survey among women aged 15–49 years in Maputo and Quelimane cities was conducted using a multi-stage clustered sampling design. Data on sociodemographic characteristics, maternal outcomes, contraceptive use, knowledge and attitudes towards the new abortion law were collected. Bivariate and multiple logistic regression analysis using the complex samples procedure in SPSS were applied.

**Results:**

A total of 1657 women (827 Maputo and 830 Quelimane) were interviewed between August 2016 and February 2017. The mean age was 27 years; 45.7% were married and 75.5% had ever been pregnant. 9.2% of the women reported having had an induced abortion, of which 20.0% (17) had unsafe abortion. Of the respondents, 28.8% knew the new legal status of abortion. 17% thought that the legalization of abortion was beneficial to women’s health. Having ever been pregnant, being unmarried, student, Muslim, as well as residing in Maputo were associated with higher odds of having knowledge of the new abortion law.

**Conclusion:**

Reports of abortion appear to be low compared to other studies from Sub-Saharan African countries. Furthermore, respondents demonstrated limited knowledge of the abortion law. Social factors such as education status, religion, residence in a large city as well as pregnancy history were associated with having knowledge of the abortion law. Only a small percentage of women perceived abortion as beneficial to women’s health. There is a need for widespread sensitization about the new law and its benefits.

## Background

Women of reproductive age are at risk for unintended pregnancy and induced abortion, especially those at young ages and living in developing countries where the access to contraception is low [1] due to financial, social and cultural barriers [1, 2]. Annually, 55.7 million abortions are taking place worldwide, of which an estimated 30,6 million are safe abortions [3], performed by a trained professional and in a suitable medical environment. The proportion of unsafe abortions, that is, the proportion of pregnancy termination carried out by persons lacking the necessary skills or in an unsuitable environment [4], is significantly higher in developing countries than in developed countries (49,5% vs 12,5%) [3].”

Mozambican women are not an exception. Maternal mortality ratio (MMR) is high - estimated at 408 deaths per 100,000 live births in Mozambique [5] - and abortion complications contribute to about 6.7% of the MMR [6]. In the 2011 Demographic Health Survey (DHS) 9.8% of women reported ever having terminated a pregnancy. Among young women aged 15–24 years, the proportion of induced abortion was 7%. DHS data from 2011 further show that 17.6% of the 13,220 reported having had births that were not wanted at that time [5].

Unpublished evidence, from the records of the Mozambican Association for Development of the Family (AMODEFA) show that between 2010 and 2016, 70,895 women of reproductive age, with unintended pregnancies, sought safe abortion services at AMODEFA clinic. Of the total, 43% were women aged 15–24 years old [7]. AMODEFA Clinic offers sexual and reproductive health services, including safe abortion. Since this figure only covers women who sought health services at that health facility, the actual number is likely to be substantially higher.

The status of abortion law has an impact on the availability of safe abortion services. Research shows that countries with enabling laws on abortion have less unsafe abortions and fewer complications linked to abortion [8]. Among countries with more liberal abortion laws, the abortion rates vary between 7 and 9 per 1000 women aged 15–44 years. While in countries where abortion law is restrictive the rates vary between 29 and 46 per 1000 women of reproductive age [8].

Mozambique is an interesting case in this perspective, as it has recently seen a shift in abortion legislation. Historically, the criminal code inherited from the colonial administration was restrictive regarding pregnancy termination. This criminal code remained after the independence until the 1980s [9–11] when the Ministry of Health issued a decree authorizing hospitals to perform abortions upon written request by a pregnant woman or guardian. Since then, the interpretation of this decree has become more flexible [9–11]. The cost of all abortion procedures (examination and treatment) was approximately USD 13.0 at a public health facility. In 2014, a more liberal abortion law was established and its guidelines were made available in 2017 [12, 13]. According to that law, women are allowed to have a legal induced abortion to unintended pregnancy under the following conditions: upon the woman’s request during the first 12 weeks of pregnancy; during the first 16 weeks if the pregnancy results from rape or incest; and during the first 24 weeks if the physical or mental health of the woman is at risk or in cases of disease or anomaly of the fetus. Women under 16 years of age or women who are not able to decide by themselves need parental or guardian consent [12, 13]. The preconditions to accessing the abortion services include a request letter, and an examination to determine the gestational age and to check for possible contra-indications for abortion.

With a liberal law in place, one would expect that the access to available safe abortion services would rapidly increase in Mozambique. However, a recent qualitative study involving young women who had experienced induced abortion found that, among those who induced abortion at a health facility, none had followed the legal procedures because they lacked information on the status of the abortion law and its procedures [14].

Recent studies on abortion in Mozambique, have focused on clinical and technical procedures for performing an abortion. These include history and physical examination with ultrasound in confirming completion of abortion [15], method preferences and experiences with misoprostol and vacuum aspiration for early abortion [16], as well as institutional barriers to access safe abortion [17]. Because most of these studies were based on hospital data they were more likely to miss out a substantial proportion of illegally induced abortions taking place outside the health facilities. Furthermore, they do not focus on community awareness of the legally available abortion services or their benefits. Thus, this paper is aimed to fill this gap by conducting a population-based study on induced abortion. The objective of this paper is to assess the level of induced abortion at the community, as well as to assess awareness of and attitudes towards the new abortion law among women of reproductive age in suburban areas of Maputo and Quelimane cities.

## Methods

### Study design and population

This paper used data from a cross-sectional survey of women of reproductive age conducted in Maputo (2016) and Quelimane (2017) cities. It uses data from 1657 women (827 in Maputo and 830 in Quelimane) taken from the main household survey conducted among women of reproductive age with the objective of understanding sexual and reproductive health. The eligibility criteria for the survey were: being a woman aged 15–49; being a resident of the study site and being a member of the selected household. Women were excluded if they did not give written consent for adult women and parental/caregiver informed consent and informed assent for women under the age of 18 and if there were no conditions for the interview to be conducted in privacy.

### Study setting

These two Mozambican cities were selected as study sites mainly because they present different social systems of family organization in Mozambique (patrilineal in Maputo vs matrilineal in Quelimane).

Maputo is the capital of Mozambique, and it is located in the South of the country. The total population at the 2017 census was 1,080,356 inhabitants of which 51.7% were women. About 56.0% of women were aged 15–49 years. Of this total, 41.3% were aged between 15 and 24 years old [18]. In Quelimane, the capital of the central province of Zambézia, the total population in 2017 was 347,907 inhabitants, of which 51.9% were women. About half (50.1%) of women were in the reproductive age (15–49 years), and 46.8% of those were aged 15–24 years [18].

Additionally, the national report from *Direcção Nacional de Planificação* of the Ministry of Health, indicated that the health facilities in Quelimane (696) and Maputo city (2629) attracted a high number of women in Gynecology Urgency, seeking post-abortion services in 2014 [19] compared to other cities located in the same region of Mozambique.

Regarding availability and access to health facilities, Maputo has 39 health facilities, of which 5 are of the level II, and 2 are of level IV. Quelimane has 12 health facilities, of which 1 is of level III, and 1 is of level IV. The remaining health facilities in both Maputo (25) and Quelimane (10) are of level I [20]. Level I and II health facilities can only offer primary care services, while health facilities of levels III and IV also offer specialized care [20]. At the moment of data collection, safe induced abortion was offered in health facilities of levels II and IV in Maputo. There was no information about safe abortion services in Quelimane.

Data on household wealth, maternal and infant mortality per city are scarce in Mozambique. Existing statistics are usually presented by area of residence (urban vs rural) and province. Thus, the flowing characteristics described are those of the provinces where the studied areas are located. The average of household expenditure per month during the period 2014/15 was 25.912,00 MZN (767.54 USD) in Maputo, while in Zambézia, where Quelimane is located, the average of expenditure per month was 3.749,00 MZN (111.04 USD [21]. Regarding reproductive health, data captured through 2011 DHS showed that in Maputo the level of infant mortality was 61 per 1000 live birth [5]; while maternal mortality was estimated in 362 per 100,000 live birth [22]. For Zambézia, this was respectively 95 per 1000 live births [5] and 508 per 100,000 births [22]. These characteristics reflect the underlying socio-economic differences between the two study sites.

### Sample size and sampling procedure

The sample size was calculated to include a representative sample of the population. The sample size was adjusted to the cluster design effect and added 5% of contingency rate to cover cases of non-response. The 50% of prevalence was used taking into account that the survey was to collect data about different sexual and reproductive health indicators such as health-seeking behaviour, gender violence, pregnancy, and induced abortion, hence resulting in the maximum sample size. A four-stage clustered sampling procedure was used to sequentially select the neighbourhoods, Enumeration Areas (EA), households and women. EA corresponds to the primary geographic unit defined for sampling in all statistics, such as census, demographic health survey, among others, developed by the National Institute of Statistics [23].

As there were a large number of neighbourhoods and limited financial resources, we used population size and socio-economic status to limit the number of neighbourhoods from which to select. All neighbourhoods with a large population (> 6000 for Quelimane and > 10,500 for Maputo) and a high proportion of the population (= > 0.56) living below the median poverty level were selected. These neighborhoods are densely populated, with difficult access, especially access to information, which is important on issues of health. The subsequent stage consisted of randomly selecting 14 households in each selected EA. This stage was followed by selecting one eligible woman for the interview in each of the selected households. For households with only one eligible woman, she was automatically selected. In households with more than one woman, the names of eligible women were listed. For a detailed description of the sampling strategy, see Additional file 1. Data were collected from August 2016 to January 2017.

### Data collection procedures

Data were collected using a questionnaire that was administered to participants in Portuguese by trained research assistants. The questionnaire was adapted from WHO questionnaires [24–26], and the 2011 Mozambican DHS [5]. Before its implementation, the questionnaire was pre-tested with 50 women of reproductive age in Quelimane and Maputo.

### Measures

The following paragraphs present the concepts used in this study and their operationalization. The main outcomes for this study were induced abortion; knowledge of the new status of abortion law, and attitude regarding the availability of abortion services at the health facility.

*Pregnancy outcomes* refer to the way in which pregnancies ended (new-born baby, spontaneous abortion or induced abortion). *Spontaneous* abortion or miscarriage as a spontaneous expulsion of the fetus due to natural causes before 28 weeks of gestation. *Induced abortion* (up to 28 weeks of gestation) [13] was defined as using any method to expel the fetus from the womb. The pregnancy outcome was capture through the questions: Have you ever been pregnant? Subsequently, we asked how many times they have been pregnant? For each pregnancy, we asked how it ended? The interviewers were trained to understand if the way pregnancy ended was forced by women, such as the use of any medicine or had been helped, or if abortion was due to natural causes, such as illness.

The reasons for pregnancy termination (induced abortion) were measured by an open-ended question that asked about the main reasons to terminate the pregnancy. The answers to this question were analyzed and grouped into 2 categories (personal and interpersonal). The intention to terminate a pregnancy refers to women who had considered an abortion while they were pregnant but did not go through with it.

*Knowledge and Attitudes towards the new legal status of abortion law* were measured through the questions: “Is induced abortion legal in Mozambique?” and “Is the legal permission of abortion at health facility beneficial for women?” (yes, no, and do not know).

*Socio-demographic* characteristics included age in years, marital status divided into two categories, married (formal or traditional union or cohabiting) vs unmarried (not in union, divorced or widow), religion (Catholic, Muslim, Protestant and “Other religion”); education level (no formal education, primary, secondary, tertiary); occupation (unemployed, employed and students).

*Knowledge of family planning and contraceptive methods* was measured through the proportion of women who have ever heard about any modern contraceptive methods and the number of contraceptive methods known. The use of contraceptive methods was measured by the proportion of women reporting having ever used contraceptives. These were measured using Yes/No questions.

### Data analysis

To allow for comparability with other studies and to ensure that the results reflect the whole target population, data were weighted based on women of reproductive age in the study area. Univariate analyses consisted of descriptive statistics such as means, frequencies, or proportions. This was followed by bivariate and multiple logistic regression analyses. All processes of data analyses either univariate, bivariate, or multiple logistic regression were performed taking into account the study design, using the complex sample procedure in SPSS version 23. First, a comparative description of participants was made, followed by bivariate analysis, to understand the association (test of independence) between the dependent and independent variables. The results were summarized in cross tables. The age was presented as mean with standard deviation (SD), while the remaining, categorical variables were presented as proportions. Multiple logistic regression analysis was applied to identify factors that explain the variability of knowledge and attitudes toward the new legal status of abortion. The covariates used in this regression to predict knowledge of and perceived benefits of abortion permission at the health facility, were: age, marital status, level of education, religion, city of residence, occupation, use of contraceptives, and experience on pregnancy. In the explanation of the perceived benefits of abortion permission at the health facility, we also added knowledge about the new abortion law as an independent variable. These variables are common in this kind of analysis, see, for example, Geleto et al. [27], Awoyemi et al. [28], Bitew et al. [29], Adinma et al. [30], Morroni et al. [31], Tedrow et al. [32]. All variables were entered at once in the model. No selection of variables was done.

The proportion of abortion among women was calculated based on, at least, one episode [33] reported by the participant.

## Results

### Description of the study participants

The main characteristics of the participants are summarized in Table 1. Of the 1657 participants, 830 (50.1%) were from Quelimane. About half 816 (51.2%) were between 15 and 24 years old. The mean age was 27 years with a SD of 9 years; 899 (53.4%) were unmarried, 1071 (63.3%) were attending or had completed secondary school, and 168 (6.8%) participants were Muslims. The analysis pointed for significant differences between the participants of the two cities in age groups [*P* < 0.05], in religion, and education level, as well as in occupation at the [*P* < 0.001], Table 1.

**Table 1:** Comparison of Socio-demographic Characteristics of women of reproductive age in Maputo and Quelimane cities

Categories	Totals	Maputo	Quelimane	
	n (%)	n (%)	n(%)	*P*-value
All cases	1657 (100)	827 (49.9)	830 (50.1)	
Mean of age		26 (9 SD)^a^	27 (8 SD)^a^	
Ages by group				0.015
15–24	816 (51.2)	444(53.6)	372 (43.4)	
25–34	503 (27.5)	226(23.9)	277 (39.4)	
35–44	255 (18.5)	115(19.6)	140 (14.7)	
45–49	75 (2.8)	42(2.9)	33 (2.5)	
Marital status				0.813
Non married	899 (53.4)	459(53.8)	440 (52.0)	
Married	758 (46.6)	368(46.2)	390 (48.0)	
Religion				0.000
Catholic	656 (25.7)	117(15.1)	539 (61.2)	
Muslim	168 (6.8)	14(1.9)	154 (23.2)	
protestant	425 (30.4)	355(37.4)	70 (6.9)	
Others	406 (37.1)	341(45.6)	65 (8.8)	
Education level				0.000
Non educated	38 (1.8)	23(1.9)	15 (1.6)	
Primary	425 (32.4)	239(36.4)	186 (18.4)	
Secondary	1071 (63.3)	533(59.7)	538(76.1)	
University	61 (2.5)	32(2.1)	29(4.0)	
Occupation				0.001
Unemployed	985 (64.8)	549(69.8)	436 (48.1)	
Student	402 (19.5)	149(16.7)	253 (28.8)	
Employed	262 (15.7)	125(13.5)	137 (23.1)	

*n* number of cases, (%) Percentage, *P*-values = Indicated the extent to which the participants of both cities were different

^a^Standard Deviation of age

### Pregnancy outcomes

In Table 2 which summarizes the pregnancy outcomes, shows that 76.5% of the surveyed women had ever been pregnant, 76.8% in Maputo and 75.5% in Quelimane. 9.2% of women who had ever been pregnant reported having experienced an induced abortion whereas 12.1% had a spontaneous abortion due to natural causes. Regarding the provider, our analyses also show that among women who induced abortion, 80.8% reported having recurred to formal health providers such as nurse and doctor, and 19.2% sought abortion at informal providers such as - by order of importance – a friend, family member or traditional healer. Regarding the place where the abortion was done, 69.6% said that it was performed at the health facility, while 30.4% had an abortion outside of a health facility. Of the latter, among women (26) reported having induced abortion out of health facility, 36.8% was done by a formal provider, and 63.2% of women in our sample received an abortion by an unskilled provider, which is, according to the WHO [2012] definition an unsafe abortion. Since the number of cases was too small, we could not do further multivariate analyses on this indicator**.** In conclusion, we can say that of the 85 participants that answered these two questions 59 (69.4%) of the abortions could be considered safe, and 17 (20.0%) unsafe. While 9 (10.6%) of abortions were performed by the formal provider, it is not possible to assess whether it was a safe or unsafe abortion, since the pregnancy was terminated outside of a health facility, in a location of which the conditions are not known.

**Table 2:** Pregnancy and outcomes among women of reproductive age in Maputo and Quelimane cities

Categories	Totals	Maputo	Quelimane	
	Weighted%	n° (%)	n° (%)	*P*-value
Pregnancy				0.787
Yes	1190 (76.5)	597 (76.8)	593 (75.5)	
No	464 (23.5)	228 (23.2)	236 (24.5)	
Induced abortion				0.024
Yes	99 (9.2)	78 (10.6)	21 (4.5)	
No	1090 (90.8)	518 (89.4)	572 (95.5)	
Spontaneous abortion^a^				0.000
Yes	140 (12.1)	105 (14.5)	35 (4.0)	
No	1049 (87.9)	491 (85.5)	558 (96.0)	
Relationship at abortion time^b^				0.170
Husband	29 (27.9)	25 (25.7)	4 (51.5)	
Boyfriend	64 (72.1)	52 (74.3)	12 (48.5)	
Ever had intention of abortion				0.020
Yes	72 (8.5)	44 (10.5)	28 (4.0)	
No	879 (91.5)	352 (89.5)	527 (96.0)	
Abortion provider				0.584
Formal provider	68 (80.8)	58 (81.5)	9 (73.1)	
Informal provider	17 (19.2)	12 (18.5)	5 (26.9)	
Place of Abortion				0.819
Health facility	59 (69.6)	49 (69.2)	10 (73.1)	
Out of health facility	26 (30.4)	21 (32.0)	5 (26.9)	

*n* number of cases, (%) Percentage, *P*-values = indicated the extent to which the participants of both cities were different

^a^relationships at abortion time was calculated only for those reported induced abortion

^b^only for those who had ever been pregnant.

Induced abortion was significantly more reported in Maputo (10.6%) than in Quelimane (4.5%). Maputo also reported a significantly higher percentage (14.5%) of spontaneous abortions than Quelimane (4.0%) (Table 2). The analysis showed statistically significant differences [*P* < 0.05] in induced abortion, and [*P* < 0.001] in spontaneous abortion, between participants of the two cities, Table 2. There was no significant difference in induced abortion between the age groups (15–24 years, 25–34 years and 35–49 years), Additional file 1.

The majority of women who had ever induced abortion (72.1%) considered their partner as a boyfriend at the time of the decision to induce abortion. The main reasons for pregnancy termination were: at the personal level- not being prepared for motherhood; already having a little child to care for; lack of financial resources to look after the baby, and at the interpersonal level or partner related reasons, refusal of paternity or not knowing who the father of the baby was.

The intention to induce abortion was reported by 8.5% of the surveyed women and differed significantly [*P* < 0.05], between the two study sites. Reasons for not pursuing the abortion intent were fear of not being able to have a baby in the future and fear of dying. Being supported by partner and family to have a baby, and lack of money to access abortion services were other factors which influenced women to continue with the pregnancy.

### Knowledge of modern contraceptives and use

The majority of the surveyed women (95.5%) had ever heard about contraceptive methods. Among these, 722 (43.6%) knew 3 or 4 modern contraceptive methods. Of those who had heard of contraceptives, 74.8% had ever used any modern contraceptive methods. More women in Maputo than Quelimane reported having ever used or currently using contraceptives (81.8% vs 71.7 and 61.9% vs 48.9% in Maputo and Quelimane respectively). The most commonly used contraceptive method was injection (Depo-Provera), being more common in Maputo (34.2%) than in Quelimane (23.7%) (Table 3). Knowledge about, as well as the use of contraceptive methods, were significantly different between the two sites at levels of [*P* < 0.01], and [*P* < 0.05] respectively. These differences appear to show that Maputo was more informed about contraceptives methods than Quelimane, Table 3.

**Table 3:** Contraceptive knowledge, uptake, and knowledge of the new abortion law among women of reproductive age in Maputo and Quelimane cities

Categories	Totals	Maputo	Quelimane	
	n (%)	n (%)	n (%)	*P*-value
Ever heard about contraceptives				0.002
Yes	1532 (95.5)	781 (96.9)	751 (90.7)	
No	112 (4.5)	36 (3.1)	76 (9.3)	
Ever Use contraceptives				0.042
Yes	1145 (79.6)	619 (81.8)	526 (71.7)	
No	386 (20.4)	161 (18.2)	225 (28.3)	
Current contraceptives usage				0.105
Yes	776 (59.2)	408 (61.9)	368 (49.8)	
No	748 (40.8)	365 (38.1)	383 (50.2)	
Contraceptive currently used				0.265
Pills				
Yes	218 (30.0)	128 (31.2)	90 (24.5)	
No	546 (70.0)	270 (68.8)	276 (75.5)	
Injection				0.143
Yes	203 (32.3)	87 (34.2)	116 (23.7)	
No	561 (67.7)	311 (65.8)	250 (76.3)	
Long term				0.168
Yes	80 (10.1)	50 (8.8)	30 (15.7)	
No	684 (89.9)	348 (91.2)	336 (84.3)	
Male condom				0.217
Yes	261 (27.6)	131 (25.6)	130 (36.1)	
No	503 (72.4)	267 (74.4)	236 (63.9)	
Modern contraceptive known				0.000
0	132 (5.6)	53 (4.6)	79 (8.8)	
1–2	341 (16.2)	108 (11.7)	233 (31.2)	
3–4	722 (41.0)	372 (40.7)	350 (42.0)	
5–7	462 (37.2)	294 (43.0)	168 (18.0)	
Knowledge of abortion law				0.000
Yes	363 (28.8)	282 (33.3)	81 (14.0)	
No	900 (43.1)	345 (36.9)	555 (63.6)	
Do not know	351 (28.1)	184 (29.8)	167 (22.4)	
Benefits of abortion permission				0.375
Yes	313 (17.3)	190 (18.6)	123 (13.0)	
No	1081 (69.2)	531 (68.8)	550 (70.4)	
Do not know	216 (13.5)	92 (12.6)	124 (16.50	

*n* number of cases, (%) Percentage; *P*-values = indicated the extent to which the participants of both cities were different

### Knowledge of the new legal status of abortion in the study site

In general, the new legal status of abortion was not well known by the majority of women studied. Only 363 women (28.8%) answered yes to the question of *whether abortion is legal in Mozambique* (33.3% in Maputo and 14.0% in Quelimane). For the question *whether the permission for abortion in the health facility is beneficial for women’s health*, 69.2% answered no (Table 3). Taken by ages, a high percentage (21.7%) of participants who said no to the question related to perceived benefits of the abortion permission was found among women at the ages 25–34 years; followed by women aged 35–49 years (18.0%), and 15.1% was the proportion of those women aged 15–24 years, see Additional file 1. There was a significant difference [*P* < 0.001] in the knowledge of the new law on abortion between Maputo and Quelimane, but not in perceived benefits. Among women with the experience of induced abortion, 34.3% reported that abortion was legal, 53.% said it was illegal and 12.5% did not know about the new status of the law, but the differences were not statistically significant, see Additional file 1.

### Factors associated with knowledge or perceived benefits of the new abortion law in the study site

On the bivariate analysis, the city of residence, education level, and use of contraceptive methods were associated with high odds of having knowledge about the new legal status of abortion law (Table 4). However, in the multiple logistic regression model, the factors that were significantly associated with knowledge of the abortion law were the city of residence (*p*-value < 0.001), the experience of pregnancy (p-value < 0.01), marital status and occupation (p-value < 0.05) (Table 4), though marital status and occupation were not significant on bivariate analysis.

**Table 4:** Bivariate and Multiple regression analysis: knowledge of new law on abortion, benefits of these services among women of reproductive age in Maputo and Quelimane cities

	Knowledge				Benefit			
	Bivariate		Multiple logistic regression		Bivariate	Multiple logistic regression		
Categories	OR	95% CI	AOR	95% CI	OR	95% CI	AOR	95% CI
City								
Maputo vs Quelimane^a^	3.05	(1.55–6.00)**	5.04	(2.75–9.24)***	1.46	(0.86–2.48)	1.42	(0.74–2.76)
Age								
25–34 vs. 15-24^a^	0.84	(0.49–1.46)	1.13	(0.54–2.35)	0.69	(0.36–1.34)	1.74	(0.85–3.56)
35–49 vs. 15–24	1.75	(0.83–3.69)	0.44	(0.16–1.20)	0.96	(0.39–2.37)	1.51	(0.54–4.23)
Religion								
Muslim vs Catholic^a^	1.77	(0.52–6.08)	0.6	(0.18–2.04)	0.22	(0.09 -0.51)***	0.22	(0.10–0.50)*
protestant vs. Catholic	0.72	(0.45–1.16)	0.69	(0.43–1.10)	0.94	(0.42 -2.10)	0.88	(0.40–1.95)
Others vs. Catholic	0.95	(0.45–2.03)	0.55	(0.29–1.06)	0.82	(0.44 -1.60)	0.78	(0.40–1.52)
Marital status								
Unmarried vs married^a^	1.74	(0.95–3.18)	2.14	(1.05–4.36)*	1.14	(0.65–2.01)	1.08	(0.64–1.80)
Education level								
Non educated vs. Secondary	3.02	(1.03–8.84)*	3.84	(0.73–20.23)	0.53	(0.15–1.85)	0.33	(0.08–1.30)
Primary vs. Secondary	0.92	(0.41–2.06)	1.04	(0.52–2.06)	0.81	(0.40–11.61)	0.78	(0.40–1.52)
University vs. Secondary	1.70	(0.78–3.67)	1.64	(0.61–4.41)	6.75	(3.33–13.69)***	6.07	(2.72–13.53)***
Occupation								
Students vs Unemployed^a^	0.77	(0.31–1.91)	2.26	(1.12–4.53)*	0.77	(0.35–1.1.68)	1.59	(0.71–3.56)
Employed vs Unemployed	0.62	(0.32–1.19)	2.02	(1.07–3.83)	0.75	(0.40–1.43)	1.06	(0.55–2.02)
Ever Use contraceptives								
Yes vs No^a^	2.17	(1.05–4.46)*	1.92	(0.97–3.83)	1.92	(1.08–3.40)*	1.46	(0.74–2.88)
Ever been pregnant								
Yes vs. No^a^	1.64	(0.83–3.23)	3.34	(1.62–6.89)**	1.34	(0.83–2.14)	1.25	(0.61–2.55)
Abortion knowledge								
Yes vs. No^a^					2.89	(1.67–5.01)***	2.54	(1.57–4.10)***

*OR* Odds Ratio, *AOR* Adjusted odds ratio, *CI* Confidence interval *P < 0.05; **P < 0.01; ***P < 0.001; ^a^Subcategory of reference

Factors associated with perceived benefits of the new abortion law on bivariate analysis were being Muslims vs Catholic, being at university vs secondary school, having an experience of contraceptives usage, as well as having knowledge about the new status of abortion law. On the multiple logistic regression model, women who were at or completed a university degree, and women who have knowledge about the new status of abortion law, both at the level of (*p*-value < 0.001), were more likely to perceive benefits from the permission to have an abortion at a health facility.

Muslim respondents were significantly more likely (*p*-value < 0.000) to report not seeing the benefits of the abortion law compared to Catholic respondents. This association showed consistence between multiple regression and bivariate analysis. The consistent odds ratio suggests a stable relationship between the independent variables and the dependent variable, regardless of the type of analysis approach.

Notably, this significant association was only observed solely among Muslims respondents. This prompts caution in interpretation, considering:

Other intersecting unstudied determinants might explain the correlation between being Muslim and not seeing benefits of the law, such as education, location of the study, socioeconomic status, etc. For example, in our study population, the majority of non-educated women were Muslims and the majority of the Muslim participants resided in Quelimane, which could potentially explain (part of) the association found.

In the survey, women were only asked about their religion and not about their level of religiosity. This could have given the study more depth to better interpret the results. For example, it is possible that the level of religiosity is the key associated factor here, and that the Muslim respondents in our study had a higher level of religiosity than the women of other religions.” instead of “Factors associated with perceived benefits of the new abortion law on bivariate analysis were being Muslims vs Catholic, being at university vs secondary school, having an experience of contraceptives usage, as well as having knowledge about the new status of abortion law. On the multiple logistic regression model, women who were at or completed a university degree, and women who have knowledge about the new status of abortion law, both at the level of (*p*-value < 0.001), were more likely to perceive benefits from the permission to have an abortion at a health facility. Muslim women were less (*p*-value < 0.05) likely to perceive the benefits of the new abortion law (Table 4).

## Discussion

The objective of this study was to estimate the proportion of induced abortion, as well as to assess the knowledge and perceived benefits of the new abortion law among women of reproductive age in Maputo and Quelimane cities. Three main lessons can be drawn for this study.

### Lesson 1:the reported proportion of women who have had an induced abortion was relatively low

From our study findings, three quarters (76.5%) of women had ever been pregnant, in both cities, Maputo (76.8%), in Quelimane (75.5%). Those pregnancies had different outcomes, and this study focused on pregnancies that ended in abortion. The percentage [9.2%; 95% CI = (6.1–13.6)] of induced abortion among women aged 15–49 years was not significantly different from 9.8% among women (aged 15–49 years) interviewed in 2011 [5]. There were also no statistically significant differences between age groups. However, the proportion of abortion found among women of reproductive age, as well as among young women is lower than the proportion of induced abortion found in other African countries. According to Guttmacher Institute report, it was estimated that 24% of pregnancies end in abortion in Southern Africa [34], where Mozambique is located. Furthermore, studies conducted in some Sub-Saharan African countries also indicated a high proportion of induced abortion, ranging from 13 to 32.8% among young women [35, 36]. The possible explanation for this could be that population-based surveys may have an internal bias as they rely on self-reported data as compared to facility-based surveys on actual occurrence of induced abortion, as such they may underestimate abortion prevalence. Underreporting abortion events can be due to the fear of being stigmatized or sanctioned by law [37]. In fact, 43.1% of the participants said that abortion was illegal and 28.1% did not know about the new legal status of abortion in Mozambique. Given that abortion is not considered legal or its new legal status is not known, women may tend not to disclose their experience due to the fear of being prosecuted by the law and morally judged by the community [37], and attributed negative labels (murderer, prostitute, women without sentiment, and irrational) which can mark them as inferiors to the ideals of womanhood [38, 39].

The majority of women who reported abortion considered their partner as a boyfriend at the time of abortion, (that is, unmarried), suggesting that the marital status may have influenced their decision. These results are similar to Sedgh et al. [40] and Awoyemi et al. [28] findings which showed that married women were less likely to demand abortion, compared to women who were not married.

As was pointed out before, in methods, the study settings differ culturally. In Maputo, children belong to the father’s family, while in Quelimane, the descendance is traced through the mother [41, 42]. For example, in matrilineal communities such as Quelimane marriage, and fatherhood is not of great importance [43], meaning that procreation does not imply “marriage”. In patrilineal Maputo, however, marriage (*lobolo – bride price)*, and fatherhood are more important. The man pays the bride price and has significant authority in decision making over a woman within marriage [44]. Differences in social organization between the study sites can, perhaps, justify the higher rates (10.6%) of induced abortion in Maputo, also supported by the association of experience on induced abortion with residence in Maputo city .

### Lesson 2: the level of knowledge on the new legal status of the abortion law in this study population is low

Generally, the law on abortion is not known. This is consistent with other studies in Ethiopia [27] and Nigeria [30] where only a third of the surveyed women were aware of the legal status of abortion. In South Africa [31] researchers found that only 32% of respondents did not know about the legal status of abortion in the country. Lack of information about the new legal status of the abortion law in these two study sites may be due to poor dissemination of the law and the fact that its guidelines were only published in September 2017, 3 years after the approval of the new law. The abortion law and its guidelines, although published, they appear to be known, fundamentally, by non-governmental organizations, such as *Rede de Defesa dos Direitos Sexuais e Reprodutivos*, which work on human rights advocacy, particularly for women. The information about abortion law and procedures is generally available at health facilities services. For example, in August 2018, the Central Hospital of Maputo published a notice stating that pregnancy termination is free and advised women to seek more information at gynaecology services [45]. The analysis showed that women who were living in Maputo city were more likely to know the abortion law compared to those at Quelimane city. Women that had experienced pregnancy were more likely to know the abortion law than women that did not experience it. Unmarried women, and being student, were also factors that increase the likelihood to know the new abortion law compared to married and employed women, respectively. Thus, women who have not been pregnant or live far away from a health facility, are “excluded” from accessing information on new abortion law because they are not directly connected to the main sources of information on sexual and reproductive health, such as prenatal care, family planning or linked to the centre of decision-making (Maputo). Married women are less likely to know the new abortion law. This is likely to be linked to their status, as procreation is of high importance to a married African woman [46]. In turn, students were more likely to know the new status of abortion law maybe because of the high promotion of sexual and reproductive health at school through the so-called *Cantinho do adolescente*. *Cantinho* is a “little corner”, established in schools across Mozambique, especially at secondary, where students receive counselling, family planning advice, and condoms. There, they can talk about gender roles as well as sexual harassment or early pregnancies [47]. Similarly, this information has been made available by other programs such as *Geração BIZ* [48], *TuaCena* [49], and *mCENAS* [50]. *TuaCena* and *mCENAS* programs use a variety of canals, particularly cell phones to interact with young people and empower them in sexual and reproductive health information through text messages. For example, young women question to *TuaCena* about abortion. It can be presumed that it is from these interactive programs, directed to young people, where students became informed about the new status of abortion law.

### Lesson 3: women in the study site do not perceive the legalization of abortion as beneficial to women’s health

The study also suggests that the community has not perceived the legalization of abortion at the health facility as beneficial to women’s health. Only 17,3% of the surveyed women agreed with this. This attitude may be due to an African belief according to which fertility and procreation are a way to ensure the continuation of the family from one generation to another. Thus, abortion can be seen as a threat to the continuation of the family [46], as well as the rejection of motherhood, seen as the essence of womanhood [46]. Muslim were less likely to perceive benefits of the abortion law, maybe because, like all other religions, this religion discourages abortion and considers this practice as sin, since a human embryo is an embryonic human being [46]. As shown in Table 4 it was among women with a high level of education and those who already knew the new legal status of the abortion law where the likelihood to see the benefits of abortion services was high. This likelihood is probably, due to the fact that for women with high educational levels or still studying it is more acceptable to plan their family and terminate a pregnancy when the time is not right to have children, and the priority is to establish their careers [51]. This could be the truth, for example, differences in the education level of participants (Maputo where 26.9% of women above 15 have completed secondary school vs 16.7% in Quelimane) could explain the higher occurrence of induced abortion between the cities [23]. In Maputo, there was a higher proportion of women who reported having experienced induced abortion compared to Quelimane. Maputo also presented a high proportion of women who knew the new status of the abortion, despite this evidence not being corroborated by perceived benefits. However, the fact that benefits of abortion permission at a health facility are perceived by those who are informed about the new status of abortion law, highlights how the lack of information is a barrier for healthy sexual and reproductive life, hence people non-informed, especially when the level of education is low, maybe unable to observe beyond their cultural and environmental context.

## Conclusions

In conclusion, the results of this study suggest limited knowledge about the new legal status of abortion law among women. This highlights the importance of informing people about it as well as raise the awareness on the condition of access and the importance of these services by explaining the reasons for the new legal status of abortion law.

In Mozambique the literature about abortion is scarce. The existing literature focuses on determinants, and factors of induced abortion, as well as the characteristics of women who induced abortion. Also, we did not find clear information about intervention, with providers and community, [52] aiming at raising awareness and support for abortion. However, non-governmental organizations are trying to do so taking the opportunity to talk about it when they have other activities with providers and communities in the context of sexual and reproductive health rights.

The results of this study suggest limited knowledge about the new abortion law among women, highlighting the importance of informing people and awareness raising on the conditions of abortion and public health and societal advantages. In Mozambique, such as in other countries, women at a certain point of life, when facing an unintended and unwanted pregnancy were considering to terminate the pregnancy. Without knowledge on the legal status of the law, the chance of searching clandestine and unsafe abortion increases, which, in turn, can also contribute to increasing the level of maternal mortality in Mozambique. It is a woman’s right to be informed about the existing reproductive health services that allow them to make an informed choice regarding the reproduction, condition for the status of complete physical, mental and social well-being in all matters relating to the reproductive system [53].

The steps recently taken by the International Federation of Gynaecology and Obstetrics (FIGO), in 2018, ordering the Needs Assessment on Safe Abortion Advocacy for the Association of Obstetricians and Gynaecologists of Mozambique [54], and the Guidelines for Activists on Safe Abortion [55] are important actions taken in the context of advocacy for safe abortion in Mozambique. Other actions that have been developed are the creation of space in schools (especially in secondary schools) where contraceptives methods and counselling are provided to adolescents; the creation of space on radios and television where adults and young people discuss and ask questions about sexuality issues. However, these actions should be also directed to the household, involving members of the same family (mother-daughter, father-son) to make it more effective.

The lack of knowledge and less perceived benefits of abortion permission at a health facility, is common in African countries, and it is related to misinterpretation of the abortion law and the negative perceptions communities hold about the abortion. Communities interpret abortion permission as encouragement of promiscuity and perceive abortion as a sinful, murderous, and abominable act [46, 56, 57]. These beliefs, together with the need of the family to maintain social dignity and avoid public censure [57], constitute pushing factor for unsafe abortion among young women, as well as the tendency of seeking abortion services without following the established procedures [14]. Studies from Ethiopia [36], Tanzania [37], South Africa [57], among others showed that women, especially adolescents still rely to unsafe methods or untrained provider to terminate pregnancy, as result of those factors mentioned above.

There is a need to expand information about the abortion law to all communities, despite recognition of the challenge to inform health professionals and women about this granted right [58]. In the majority of sub-Saharan countries abortion still restrictive; increasingly adolescents are becoming sexually active at an early age, without being well informed on the precaution they need to take to prevent pregnancy. Considering this scenery of early sexual activity, more research is needed to understand the impact of poor knowledge about abortion law and existing services demanding health care services among women of reproductive age.

### Study limitations

This study had a few limitations. First, since the data for this study were collected in two cities the results cannot be generalized for the whole country because of the differences in social structures, although they can help to understand how informed these specific communities are. Further, we acknowledge that we did not use internationally validated measures to measure abortion attitudes. However, the study tool was pretested for clarity and consistency. Finally, this study showed several differences in sociodemographic characteristics between the two cities and those differences could explain the negative confounding observed in Table 4. This is a cross-sectional study and cannot fully determine causality between sociodemographic characteristics on the one hand and knowledge of new abortion law, and perceived benefits of abortion permission on the other hand. We recognize this limitation and we recommend future studies to include other possible confounding variables.

### Supplementary information

**Additional file 1.**

## Data Availability

Dataset is part of a broader research for the fulfilment of the requirements for the degree of Doctor of Philosophy of the first author. It may be shared, if necessary, on reasonable request.

## Abbreviations

AMODEFA: Mozambican Association for Development of the Family
DHS: Demographic Health Survey
EA: Enumeration Areas
FIGO: International Federation of Gynaecology and Obstetrics
Geração BIZ (busy generation): Nacional Program to Improve Adolescent and Youth Sexual and reproductive health
CI: Confidence interval
mCENAS: Mobile Phone Programs for Adolescent and Youth Sexual and Reproductive Health
MMR: Maternal Mortality Ratio
MZN: Mozambique New Metical. Metical is the official currency of Mozambique
OR: Odds Ratio
AOR: Adjusted odds ratio
SD: Standard deviation
SPSS: Statistical Package for the Social Science
TuaCena: Communication for Health program for Adolescent and Youth
USD: United States Dollar. Dollar is the official currency of the United States of America
WHO: Word health organization
