# Correction: Induced abortion: a cross-sectional study on knowledge of and attitudes toward the new abortion law in Maputo and Quelimane cities, Mozambique

**DOI:** 10.1186/s12905-024-03152-6

**Published:** 2024-06-21

**Authors:** Mónica Frederico, Carlos Arnaldo, Peter Decat, Adelino Juga, Elizabeth Kemigisha, Olivier Degomme, Kristien Michielsen

**Affiliations:** 1 Centro de Estudos Africanos, Eduardo Mondlane University, Maputo, Mozambique; 2 International Centre for Reproductive Health (ICRH), Department of Public Health and Primary Care, Faculty of Medicine and Health Sciences, Ghent University, Ghent, Belgium; 3 Department of Family Medicine and Primary Health Care, Ghent University, Ghent, Belgium; 4 Department of Mathematics and Informatics, Faculty of Sciences, Eduardo Mondlane University, Maputo, Mozambique; 5 I-BioStat, Hasselt University, Diepenbeek, Belgium; 6 Faculty of Interdisciplinary Studies, Mbarara University of Science and Technology, Mbarara, 1410 Uganda

**BMC Women’s Health (2020) 20:129.**

10.1186/s12905-020-00988-6.

Following publication of the original article [1], the author noticed the errors in Table 4, and the text part in the Results section.

In Table 4, under Benefit (Bivariate and Multiple logistic regression) column, the values are published incorrectly in Religion sub-headings have been corrected as shown below:

In the *Results* section, under the subheading *Factors associated with knowledge or perceived benefits of the new abortion law in the study site,* the paragraph should read as “Factors associated with perceived benefits of the new abortion law on bivariate analysis were being Muslims vs Catholic, being at university vs secondary school, having an experience of contraceptives usage, as well as having knowledge about the new status of abortion law. On the multiple logistic regression model, women who were at or completed a university degree, and women who have knowledge about the new status of abortion law, both at the level of (*p*-value < 0.001), were more likely to perceive benefits from the permission to have an abortion at a health facility.

Muslim respondents were significantly more likely (*p*-value < 0.000) to report not seeing the benefits of the abortion law compared to Catholic respondents. This association showed consistence between multiple regression and bivariate analysis. The consistent odds ratio suggests a stable relationship between the independent variables and the dependent variable, regardless of the type of analysis approach.

Notably, this significant association was only observed solely among Muslims respondents. This prompts caution in interpretation, considering:

Other intersecting unstudied determinants might explain the correlation between being Muslim and not seeing benefits of the law, such as education, location of the study, socioeconomic status, etc. For example, in our study population, the majority of non-educated women were Muslims and the majority of the Muslim participants resided in Quelimane, which could potentially explain (part of) the association found.

In the survey, women were only asked about their religion and not about their level of religiosity. This could have given the study more depth to better interpret the results. For example, it is possible that the level of religiosity is the key associated factor here, and that the Muslim respondents in our study had a higher level of religiosity than the women of other religions.” instead of “Factors associated with perceived benefits of the new abortion law on bivariate analysis were being Muslims vs Catholic, being at university vs secondary school, having an experience of contraceptives usage, as well as having knowledge about the new status of abortion law. On the multiple logistic regression model, women who were at or completed a university degree, and women who have knowledge about the new status of abortion law, both at the level of (*p*-value < 0.001), were more likely to perceive benefits from the permission to have an abortion at a health facility. Muslim women were less (*p*-value < 0.05) likely to perceive the benefits of the new abortion law (Table 4).”

The original article has been corrected.

**Table 4:** Bivariate and multiple regression analysis: knowledge of new law on abortion, benefits of these services among women of reproductive age in Maputo and Quelimane cities

	Knowledge				Benefit			
	Bivariate		Multiple regression		Bivariate		Multiple regression	
Categories	OR	95% CI	AOR	95% CI	OR	95% CI	AOR	95% CI
City								
Maputo vs. Quelimane^a^	3.05	(1.55-6.00)**	5.04	(2.75–9.24)***	1.46	(0.86–2.48)	1.42	(0.74–2.76)
Ages								
25–34 vs. 15-24^a^	0.84	(0.49–1.46)	1.13	(0.54–2.35)	0.69	(0.36–1.34)	1.74	(0.85–3.56)
35–49 vs. 15–24	1.75	(0.83–3.69)	0.44	(0.16–1.20)	0.96	(0.39–2.37)	1.51	(0.54–4.23)
Religion								
Muslim vs. Catholic^a^	1.77	(0.52–6.08)	0.6	(0.18–2.04)	0.22	(0.09–0.51)***	0.22	(0.10–0.50)*
Protestant vs. Catholic	0.72	(0.45–1.16)	0.69	(0.43–1.10)	0.94	(0.42–2.10)	0.88	(0.40–1.95)
Others vs. Catholic	0.95	(0.45–2.03)	0.55	(0.29–1.06)	0.82	(0.44–1.60)	0.78	(0.40–1.52)
Marital status								
Unmarried vs. married^a^	1.74	(0.95–3.18)	2.14	(1.05–4.36)*	1.14	(0.65–2.01)	1.08	(0.64–1.80)
Education level								
Non educated vs. Secondary	3.02	(1.03–8.84)*	3.84	(0.73–20.23)	0.53	(0.15–1.85)	0.33	(0.08–1.30)
Primary vs. Secondary	0.92	(0.41–2.06)	1.04	(0.52–2.06)	0.81	(0.40-11.61)	0.78	(0.40–1.52)
University vs. Secondary	1.70	(0.78–3.67)	1.64	(0.61–4.41)	6.75	(3.33–13.69)***	6.07	(2.72–13.53)***
Occupation								
Students vs. Unemployed^a^	0.77	(0.31–1.91)	2.26	(1.12–4.53)*	0.77	(0.35–1.1.68)	1.59	(0.71–3.56)
Employed vs. Unemployed	0.62	(0.32–1.19)	2.02	(1.07–3.83)	0.75	(0.40–1.43)	1.06	(0.55–2.02)
Ever Use contraceptives								
Yes vs. No^a^	2.17	(1.05–4.46)*	1.92	(0.97–3.83)	1.92	(1.08–3.40)*	1.46	(0.74–2.88)
Ever been pregnant								
Yes vs. No^a^	1.64	(0.83–3.23)	3.34	(1.62–6.89)**	1.34	(0.83–2.14)	1.25	(0.61–2.55)
Abortion knowledge								
Yes vs. No^a^					2.89	(1.67–5.01)***	2.54	(1.57–4.10)***

Odds Ratio *P < 0.05; **P < 0.01; ***P < 0.001; ^a^Subcategory of reference
