# Supplementary material for: Induced abortion: a cross-sectional study on knowledge of and attitudes toward the new abortion law in Maputo and Quelimane cities, Mozambique
Source: BMC Womens Health. 2020 Jun 19;20:129. doi: 10.1186/s12905-020-00988-6 (PMC7304116; doi:10.1186/s12905-020-00988-6)
Supplement: Supplementary file 1 — Additional file 1. [file 12905_2020_988_MOESM1_ESM.docx]

**Appendix 1- Description of the strategy used to select participants**

- Stage 1: Neighbourhoods. All neighbourhoods with a large population (>6 000 for Quelimane and >10 500 for Maputo)^1^ and a high proportion (=> 0.56) of the population living below the median poverty level were selected. In total, 25 neighbourhoods were included. Given the fact that the flow of and quality of the information in these areas is low, working in these areas was the best strategy for assessing the level of knowledge of abortion law and related attitudes, as well as evaluating the occurrence of unsafe or illegal abortion.
- Stage 2: EA: 60 EAs were randomly selected in both Maputo and Quelimane. The determination of the number of clusters (EAs) per neighbourhood were based on the population size.
- Stage 3: Households. In each selected EA 14 households were randomly selected and the number of households was determined based on existing studies that indicate the optimal number of households in each cluster, varying from 10 to 16^2^.
- Stage 4: Women. One eligible woman was selected for the interview in each of the selected households. For households with only one eligible woman, she was automatically selected. In households with more than one woman, names of eligible women were listed and attributed a number which was used to select one of them randomly, using the App holo random number generator. Data were collected from August 2016 to January 2017.

^_______________________________________^

^1^ Ministério de Economia e Finanças. Pobreza e Bem-Estar em Moçambique: Quarta Avaliação Nacional (Iof 2014/15). Maputo, Moçambique. 2016. Http:// <https://www.wider.unu.edu/sites/default/files/Final_QUARTA>. Accessed:07.10.2017.

^2^Cubula BS. Os Erros não Amostrais e os Fatores da Não-Resposta em Inquéritos por Amostragem na Cidade De Maputo. Dissertação de Mestre. Instituto Superior de Estatística e Gestão de Informação Universidade Nova de Lisboa. 2013. Https://run.unl.pt/bitstream/10362/10497/1/TEGI0342.pd. Accessed 12.03.2019.

**Appendix 2. Pregnancy outcomes, knowledge of and attitudes toward abortion law by group of age among women of reproductive age in Maputo and Quelimane cities**

|  | Groups of age | | | | | | |
| --- | --- | --- | --- | --- | --- | --- | --- |
|  |  | 15-24 |  | 25-34 |  | 35-49 |  |
| Categories | n | % | n | % | n | % | *P* -value |
| Ever induced abortion |  |  |  |  |  |  | 0.071 |
| Yes | 36 | 5.8 | 40 | 15.0 | 23 | 6.9 |  |
| No | 371 | 94.2 | 416 | 85.0 | 296 | 93.1 |  |
| Abortion law |  |  |  |  |  |  | 0.163 |
| Yes | 159 | 30.1 | 132 | 33.8 | 72 | 19.8 |  |
| Do not know | 643 | 69.9 | 353 | 66.2 | 247 | 80.2 |  |
| Abortion permission |  |  |  |  |  |  | 0.056 |
| Yes | 63 | 15.1 | 102 | 21.7 | 70 | 18.0 |  |
| No | 268 | 60.4 | 289 | 66.8 | 210 | 77.8 |  |
| Do not know | 66 | 24.5 | 52 | 11.5 | 33 | 4.2 |  |

n-number of participants;% percentage; *P*-v level of significance

**Appendix 3.** **Proportion of induced abortion by knowledge of new status of abortion law among women of reproductive age in Maputo and Quelimane cities**

|  | Ever induced abortion | |  |
| --- | --- | --- | --- |
|  | n | % | *P-*value |
| Knowledge of abortion law |  |  | 0.131 |
| Yes | 38 | 34.3 |  |
| No | 45 | 53.2 |  |
| Do not know | 16 | 12.5 |  |

n-number of participants; % percentage; *P*-v level of significance
